# Environmental drivers of tick density in UK dairy farms: implications for livestock health and agri-environment policy

**DOI:** 10.1186/s13071-026-07345-w

**Published:** 2026-03-31

**Authors:** Sarah Shanks, Jennifer Duncan, Nicholas Johnson, Jake Goswell, Catherine Hartley, Richard Hassall, Bethan V. Purse, Caroline Millins

**Affiliations:** 1 Department of Livestock and One Health, Institute of Infection, Veterinary and Ecological Sciences, University of Liverpool, Liverpool, UK; 2 Vector Borne Diseases, Virology Department, Animal and Plant Health Agency, Woodham Lane, Addlestone, UK; 3 Department of Infection Biology and Microbiomes, Institute of Infection, Veterinary and Ecological Sciences, University of Liverpool, Liverpool, UK; 4 UK Centre for Ecology and Hydrology, Benson Lane, Crowmarsh Gifford, Wallingford, Oxfordshire UK

## Abstract

**Background:**

Ticks are important vectors of livestock and human pathogens in Europe. Environmental policies promoting woodland creation and habitat restoration are increasing habitat suitability for *Ixodes ricinus* but impacts on livestock tick-borne disease risk remain unclear. This study examined how landscape features influence tick distribution on UK dairy farms with a recent history of tick-borne disease.

**Methods:**

Questing ticks were sampled on 72 pastures in 12 dairy farms in southwest England (2376 transects), stratified by distance from pasture boundaries and adjacency to woodland or non-woodland habitats. Environmental variables were measured at transect, boundary, and pasture scales. Generalized linear mixed models identified predictors of tick presence in pastures, and nymph density at pasture boundaries. Farm-level associations between tick abundance, woodland cover, and cattle pathogen prevalence were assessed descriptively.

**Results:**

A total of 1701 ticks were collected (91.3% nymphs). Ticks were detected on 20% of transects and in 89% of pastures, with densities strongly aggregated at pasture boundaries. The proportion of woodland cover within 50 m buffers was the dominant environmental driver at both boundary and pasture scales, with greater cover associated with higher nymph densities and increased probability of tick presence. Boundaries adjacent to water also supported significantly higher nymph densities.

**Conclusions:**

Local landscape features, particularly woodland cover and small water bodies at boundaries, strongly influence tick distribution in UK dairy pastures. Woodland expansion through environmental schemes may therefore be associated with increased tick distribution and densities in farmed landscapes, with implications for livestock exposure and public health.

**Graphical Abstract:**

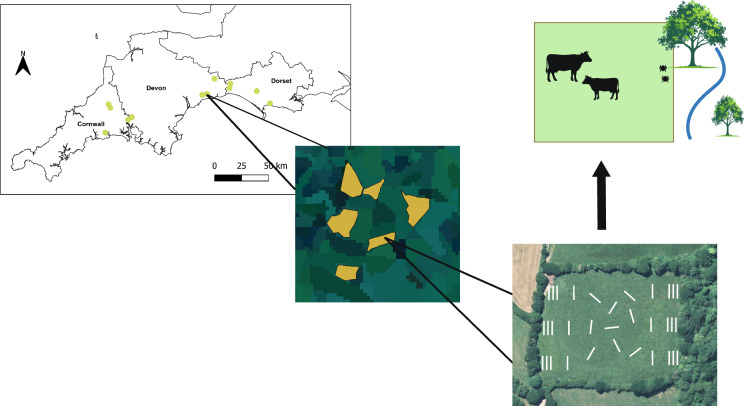

**Supplementary Information:**

The online version contains supplementary material available at 10.1186/s13071-026-07345-w.

## Background

The distribution of vector-borne diseases is changing globally, with significant implications for human and animal health. These changes have been associated with multiple interacting factors that influence vector, pathogen, and host dynamics, including climate change, land use change, and the encroachment of agriculture into natural ecosystems [1–4]. In turn, environmental governance and land management policies are shaping land use transitions, raising concerns that policy-driven landscape change may inadvertently contribute to the emergence and spread of vector-borne diseases [5, 6].

Ticks are among the most important arthropod vectors in Europe, transmitting multiple pathogens of both medical and veterinary concern. *Ixodes ricinus*, the most widespread tick species on the continent, is responsible for the highest burden of tick-borne disease in humans and livestock [7, 8]. The abundance and distribution of *I. ricinus* varies greatly across different landscapes, reflecting a dependence on suitable environmental conditions and availability of hosts. Microclimate, including a relative humidity of greater than 80% for off-host developmental phases, and the availability of vertebrate hosts are both strongly influenced by habitat and landscape structure [9–12]. Small mammals serve as the primary hosts for nymphs and larvae [13], while adult ticks feed most frequently on large herbivores such as deer and cattle. Densities of *I. ricinus* are commonly highest in woodland and ecotonal habitats that support the requisite microclimate and host abundance, but can also survive in open areas such as rough grasslands if conditions are favorable [14–17].

Across Europe, environment and climate policies promoting afforestation, natural regeneration, and habitat connectivity, such as the EU Biodiversity Strategy for 2030 [18], are implicated in creating macroclimatic and local microclimatic conditions favorable for *I. ricinus* survival and driving increases in tick populations through changes in wildlife host distributions [19–21]. Transitions toward sustainable agricultural practices are also central to these strategies, aligning with global initiatives to integrate biodiversity conservation and restoration into farming systems [22]. Although the biodiversity and climate benefits of these landscape changes are widely acknowledged, their implications for livestock health remain poorly understood, particularly where altered habitats are likely to increase proximity between livestock, wildlife, and tick vectors.

For cattle, the two most important tick-borne diseases in northern Europe are bovine babesiosis, primarily caused by *Babesia divergens*, and tick-borne fever, caused by *Anaplasma phagocytophilum*, both transmitted by *I. ricinus*. Babesiosis is recognized as an emerging infectious disease in Europe, with outbreaks linked to expansion of the vector range and changing host dynamics [23]. In adult cattle, disease can be severe, characterized by fever, hemoglobinuria and depression, and often fatal [24]. Outbreaks, often associated with previously unexposed cattle or sporadic exposure to infection, result in significant losses [25, 26]. Tick-borne fever is rarely fatal but contributes to considerable production losses through reduced milk yield, abortions, and stillbirths [27]. Despite these impacts, the environmental and farm-level drivers of the diseases remain poorly characterized. A small number of studies have reported associations between woodland proximity, ecotonal habitats, and tick abundance in cattle pastures in France [28–30]. However, these effects are determined by complex dynamics among habitat structure, host community composition, and local microclimatic conditions. Further research is needed to understand how these factors influence vector–host pathogen dynamics across different landscapes and agricultural systems.

The UK dairy sector provides a valuable case study in this context. Evidence suggests the distribution of *I. ricinus* is changing in the UK [31], and tick-borne pathogens are being detected in new areas [32]. Tick-borne diseases rank among the top three health priorities for extensively grazed livestock in the UK, and are considered as “high-impact, low-prevalence” conditions by the farming industry [33, 34]. At the same time, national policy aims to establish nearly 1 million hectares of new woodland by 2050, with farmers expected to deliver much of this expansion through agri-environment schemes that incentivize the creation of wildlife-rich habitats [35].

This study investigates the landscape-scale environmental factors influencing the abundance and distribution of ticks on grazing pastures within dairy farms with a recurrent history of bovine babesiosis or tick-borne fever in the herd in southwest England. We distinguish between within-pasture micro-climate and vegetation effects and those associated with ecotonal habitats and woodland proximity surrounding pastures. A parallel study conducted on the same farms examined the prevalence of subclinical infections with *B. divergens* and *A. phagocytophilum* in the cattle and is reported elsewhere [80] . Here we leverage that data to examine broad-scale correlations between farm landscape features, tick abundance, and pathogen prevalence in cattle. Finally, we consider the implications of our findings in the context of current agricultural and environmental policies driving landscape change, as well as their relevance for disease control in both livestock and humans.

## Methods

### Farm selection

**Fig. 1:**
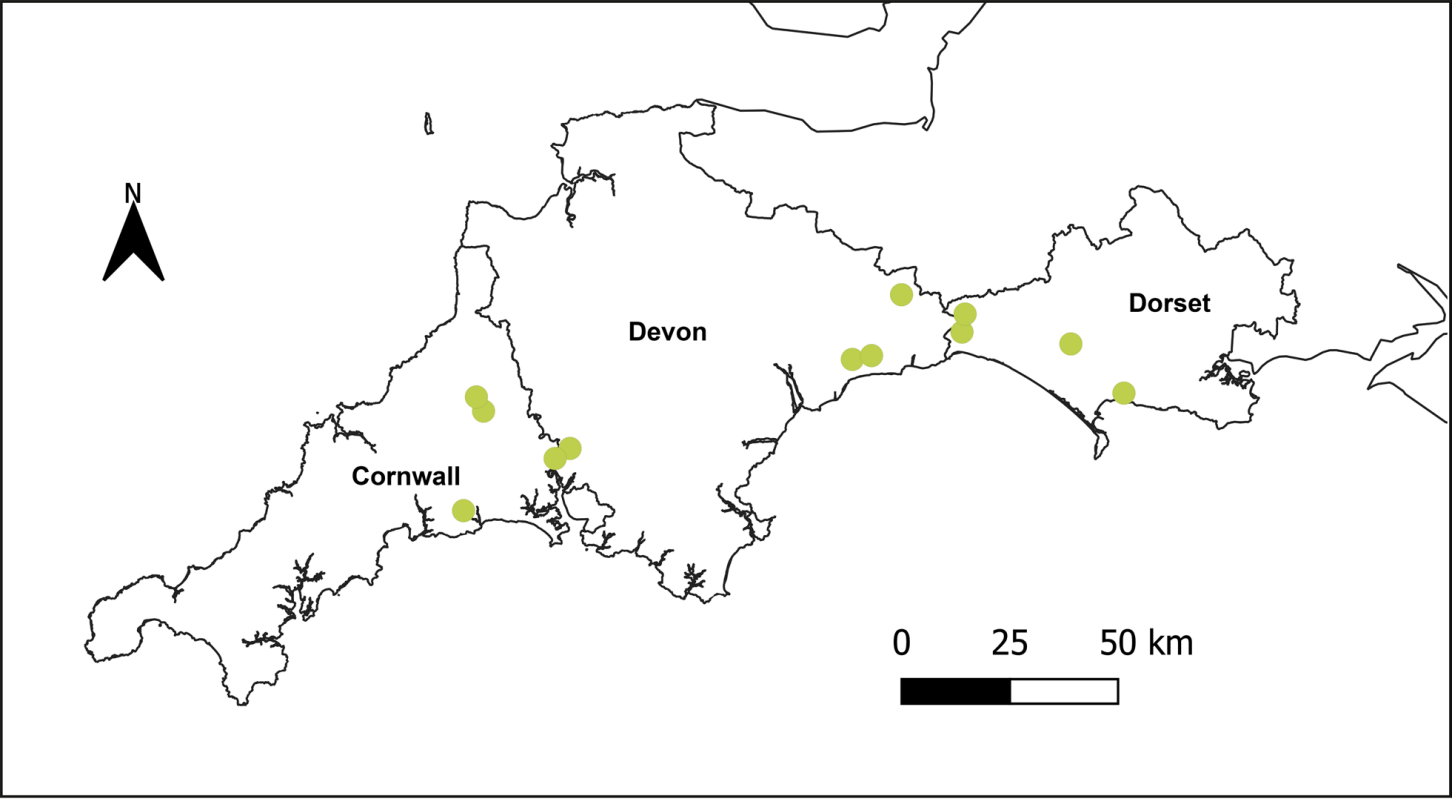
Map of southwest England showing outlines of the three counties included in the study (Cornwall, Devon, and Dorset). Farm locations are indicated in green

A total of 12 dairy farms were purposively recruited from the counties of Cornwall, Devon, and Dorset in southwest England through local veterinary networks (Fig. 1) to ensure broadly comparable grazing and management systems across study sites. This region accounts for approximately 33% of the national herd and 39% of dairy cattle specifically [36], and has an established presence of *I. ricinus* and tick-borne disease in cattle [37–40]. The area is predominantly rural, characterized by agricultural land, woodland, heathland, and coastal habitats that support a mixture of livestock farming systems including beef and dairy cattle [36, 41]. Farms were eligible for inclusion if they (i) did not routinely purchase replacement stock, (ii) had a veterinary diagnosed history of recurrent bovine babesiosis and/or tick-borne fever in the herd, and (iii) maintained a minimum of 50 breeding cows.

### Farm characteristics

Preliminary discussions were conducted with farmers to gather information on general grazing and management practices, and to identify the pastures used for cattle grazing. Herd sizes ranged from 95 to 400 cattle. All farms grazed the milking herd on pasture for at least part of the day or night from spring to autumn and housed them indoors during the winter months. Only dairy cattle were present on the study farms; no beef cattle were kept; this was not an explicit inclusion criterion but reflected the characteristics of the recruited farms. Four farmers reported winter grazing of sheep, but specific information on which pastures and when grazing occurred was not available. All farmers reported regular deer presence, including sightings in cattle grazed pastures. Acaricides were used for tick control on all but one farm, however, none are licensed for this purpose in cattle in the UK and their use was therefore off-label.

### Sampling strategy

A sampling framework was designed to capture variation in tick abundance across environmental gradients, incorporating pasture characteristics, boundary features, and fine-scale microhabitat conditions at drag locations.

To investigate variability in tick distribution across a landscape gradient, the pastures for sampling were selected on the basis of pasture grazing history and proximity to woodland. Specifically, pastures grazed by cows within the previous 12 months were categorized by whether a pasture boundary bordered woodland or not. Six pastures per farm were selected, three with a woodland border if available, and the remainder from non-woodland border pastures. Within each selected pasture, boundaries with the most and least dense bordering woodland were chosen for sampling; where no difference was apparent, two boundaries were randomly selected.

**Fig. 2:**
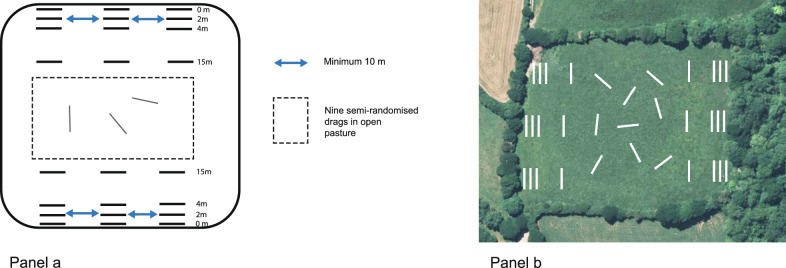
Schematic of within-pasture sampling design (**a**) and example field showing transect (white bars) placement (**b**). Distances are not to scale. Background imagery: Google Earth with the author modifications

Tick sampling was conducted using the blanket-dragging approach [42]. A 1 m^2^ white blanket was pulled slowly across the ground in 10 m transects. After each transect, the blanket was inspected, and all life stages of ticks were counted and removed. At each selected pasture boundary, three sets of four standardized drags were carried out, stratified by distance from the boundary: 0 m (immediately adjacent to the physical boundary feature such as hedgerow, fence, or ditch, *n* = 3), 2 m (*n* = 3), 4 m (*n* = 3), and 15 m (*n* = 3), with the latter representing semi-open pasture. An additional nine semi-randomized drags were carried out in each pasture at a distance greater than 15 m from all boundaries to represent open pasture, with an intended minimum spacing of approximately 10 m between transects, although complete spatial separation could not always be guaranteed in smaller fields. The three sets of drags stratified by distance from the boundary were separated by at least 10 m (Fig. 2). This design was used to capture spatial variation in tick presence relative to pasture edges, because ticks have been found to be concentrated at edges of grazing pastures in a prior study from France [29]. Sampling took place in May and June 2024, with all drags in a given pasture completed on the same day under dry weather conditions. Tick sampling was conducted by the lead researcher across all farms, with assistance from a trained student on a small number of farms, following the same standardized sampling protocol. Sampling was carried out during daylight hours; time of day was not explicitly standardized or included as a covariate in analyses. Questing adults and nymphs were collected from the vegetation. In total, 33 drags were carried out per pasture, giving 2376 drags across the study.

Questing ticks were assumed to be *I. ricinus* on the basis of previous surveillance, confirming this as the predominant questing species in southern England [38]. *Dermacentor reticulatus* has also been found in the region [43], however, any questing *Dermacentor* spp. would have been adults, readily distinguished by their larger size and ornate scutum. The nymphs of *D. reticulatus* are reportedly nidicolous, whereas adults exhibit exophilic behavior and are the only stages likely to be collected by blanket-dragging [44]. To further assess whether other Ixodes species were present among the sampled nymphs, a subset was screened using 16S rRNA polymerase chain reaction (PCR) and sequencing, with all successfully typed specimens identified as *I. ricinus* (see Supplementary Methods).

### Environmental predictors

A hierarchical framework was used to investigate environmental factors driving tick presence/absence and nymph density across three nested spatial scales: transect scale (microhabitat), pasture boundary scale (ecotonal habitat), and pasture scale (landscape context) (Table 1).

**Table 1:** Environmental variables measured at three spatial scales to investigate tick presence/absence and nymph density. Subcategories for each variable are listed

Scale	Variable	Subcategories	Summary values across all transects (*n* = 2376)		
			Mean ± SD	Range	Frequency (%)
Transect	Location relative to pasture boundary	0 m, 2 m, 4 m, 15 m, open pasture	–	–	–
	Vegetation height (cm)	–	17.96 ± 12.81	0–100	–
	Vegetation density (score 0–100)	–	7.35 ± 7.78	0–65	–
	Number of cattle dung piles	–	0.54 ± 1.08	0–8	–
	Dominant vegetation type (% of transects)_a_	Grass Herbaceous/wildflowers Bare ground Bramble/fern Rushes	–	–	92.0 4.1 1.7 1.5 0.7
Boundary	Structural boundary elements (present/absent)_b_	Hedgerow Visible fence Ditch	–	–	81.0 61.9 5.3
	Dominant habitat type within 10 m outside pasture boundary (% of transects)_c_	Pasture Trees Track/road Scrubland Water	–	–	47.5 26.3 18.3 4.2 3.7
	Woodland within 50 m buffer (%) (boundary)	–	8.79 ± 15.20	0–70	–
Pasture	Saturation deficit	–	6.90 ± 2.33	2.30–13.94	–
	Woodland within 50 m buffer (%) (entire pasture)	–	16.22 ± 16.48	0–61	–

_a_ Up to three vegetation types were initially recorded for each transect as % cover. For analysis, only the single dominant type was retained as a categorical variable

_b_ Structural elements were recorded as present/absent; multiple could be present per recording

_c_ Habitat type within 10 m of the pasture boundary was recorded as a single category (pasture, woodland, track/road, scrubland, or water)

At the finest resolution, vegetation type, height, and density were recorded for each transect to characterize microhabitat features influencing tick survival and questing behavior [16, 45–47]. Ground vegetation height and density were measured once at the end of each transect using a sward stick, with density expressed as the number of 5 cm increments on the stick obscured by vegetation. The three dominant vegetation types and their proportions were recorded for each transect, categorized into five classes: (i) herbaceous plants and wildflowers, (ii) bracken and bramble, (iii) rushes, (iv) grass, and (v) bare ground. Cattle dung piles were counted within each transect to provide an index of cattle abundance, following approaches applied to other host species [48]. Cattle can redistribute ticks across pastures through movement, potentially influencing local tick distribution patterns [46]. In addition, dung piles could influence tick survival and questing activity by physically covering or obstructing ticks.

At the pasture boundary scale, structural and contextual features hypothesized to influence tick abundance along pasture edges were recorded. Pasture boundaries were classified by the presence or absence of structural elements (hedgerow, visible fence, ditch) that may provide refuges or pathways for tick hosts [28] or modify micro-climate conditions; more than one element could be present in the same boundary. The dominant habitat within 10 m on the opposite side of the boundary at the location of 0 m transects was recorded as a single observed category (pasture, trees, water body, scrubland, track/road) on the basis of direct field observation of the visually predominant habitat type. This variable is hereafter referred to as the adjacent habitat type and represents the immediate landscape context influencing ecotonal conditions and host behavior [29, 30]. The “trees” category included both isolated trees and boundaries adjoining larger woodland patches.

To assess broader landscape influences, the proportion of woodland cover (coniferous and deciduous) was quantified within a 50 m buffer at two spatial scales: (i) around individual pasture boundaries and (ii) around entire pastures. The 50 m buffer was chosen to capture the effect of surrounding woodland [30, 49], while minimizing dilution by more distant open habitats and overlap between neighboring pastures. Data on land cover were extracted from the UK Centre for Ecology and Hydrology Land Cover Map (tiff 2022) [50] using R version 4.4.1[51] and QGIS version 3.40 Bratislava [52].

At the pasture scale, saturation deficit was included as a climatic variable influencing tick desiccation risk and questing activity [53]. Temperature and humidity were measured at vegetation height immediately before the first drag and after the last drag in each pasture using a Reed R1910 temperature and humidity logger, with the same instrument used throughout the study. Saturation deficit was calculated from temperature and humidity using the formula: ${\mathrm{SD}}\, = \,\left( {{1}\, - \,{{{\mathrm{RH}}} \mathord{\left/ {\vphantom {{{\mathrm{RH}}} {{1}00}}} \right. \kern-0pt} {{1}00}}} \right)\, \times \,{4}.{9463}\, \times \,{\mathrm{e}}^{{(0.0{621} T)}}$ where *T* is temperature in °C and *RH* is relative humidity (%) [54].

### Statistical analysis

All statistical analyses were performed using RStudio 2024.09.01. Generalized linear mixed models (GLMMs) were fitted using the glmmTMB package [55] to investigate the influence of environmental factors on tick presence/absence and nymph density. Model assumptions, including overdispersion, zero-inflation, and outliers were assessed using simulation-based residuals from the DHARMa package [56]. Collinearity among fixed effects was checked using variance inflation factors (VIF) and model convergence was verified.

### Environmental factors influencing tick presence at transect level

To examine environmental effects on the presence or absence of questing ticks (nymphs and adults), a binomial GLMM with logit link was fitted to the full dataset of 2376 transects. At the transect level, fixed effects included vegetation height, vegetation density, distance from pasture boundary, dominant vegetation type, and the number of cow dung piles. At the pasture level, fixed effects included saturation deficit and the proportion of woodland in a 50 m pasture buffer. No boundary-level predictors were included in this model. Random intercepts were fitted for farm (*n* = 12) and pasture (*n* = 72). Backward stepwise model selection was performed using the drop1 function, retaining predictors on the basis of minimization of Akaike information criterion (AIC) [57], with predictors retained where their removal resulted in an increase in AIC and models differing by ΔAIC < 2 considered to have comparable support. The relative contribution of retained predictors was assessed from their effect sizes, confidence intervals, and changes in AIC. Model performance was evaluated using conditional (including random effects) and marginal (fixed effects only) area under the receiver operating characteristic curve (AUC) values, calculated with the pROC package in R [58] and the true skill statistic (TSS) [59].

### Environmental factors influencing nymph density at pasture boundaries

To identify environmental risk factors and pasture boundary characteristics associated with nymph density, a negative binomial GLMM was fitted to nymph counts recorded on 0 m transects. At the transect level, fixed effects included vegetation height, vegetation density, dominant vegetation type, and number of cow dung piles. At the boundary level, fixed effects included presence or absence of structural boundary features, the adjacent habitat type, and the proportion of woodland in a 50 m boundary buffer. At the pasture level, saturation deficit was included. Random intercepts were fitted for farm (*n* = 12), pasture (*n* = 72), and boundary (*n* = 144). The same AIC-based backward stepwise selection was applied. Model fit was assessed using marginal and conditional *R*^2^ [60] and root mean squared error (RMSE) based on conditional (including random effects) and marginal (fixed effects only) predictions.

Two-way interaction terms between the proportion of woodland in a 50 m boundary buffer and the adjacent habitat type were also evaluated to test whether effects of woodland differed by adjacent habitat. Competing models with and without these interactions were compared using AIC and marginal and conditional *R*^2^ values, calculated with the MuMIn package [61].

### Cattle pathogen prevalence and associations with tick abundance

Broad-scale, farm-level associations between cattle *A. phagocytophilum* and *B. divergens* prevalence, mean tick abundance, and woodland cover across sampled grazing pastures per farm were examined using Spearman rank correlation coefficients (*ρ*). Herd-level prevalence data for adult cattle were obtained from cross-sectional blood sampling of cattle conducted on the same farms during the same grazing year as tick sampling. Blood samples were collected from adult cows on 12 dairy farms between September and October 2024, following the main period of tick activity in the UK. Sample sizes per farm ranged from 25 to 76 adult cattle, depending on herd size, giving a total of 741 adult cows included in the prevalence analysis. Whole blood samples were tested using multiplex real-time polymerase chain reaction (PCR) assays targeting *A. phagocytophilum* and *B. divergens*. Farm-level prevalence was calculated as the proportion of sampled adult cattle testing PCR-positive for each pathogen and was used here for descriptive, farm-level comparisons with mean tick abundance and landscape characteristics. Full methodological details of the cattle prevalence study are reported elsewhere[80  ]. For this analysis, PCR results were summarized as the proportion of adult cattle per farm testing positive for *A. phagocytophilum* and *B. divergens*. Mean tick abundance was calculated as the mean number of ticks per 0 m transect per farm. At the farm level, woodland cover was summarized as the mean proportion of woodland within 50 m buffers around all sampled pastures.

## Results

### Distribution of questing ticks within dairy pastures

**Fig. 3:**
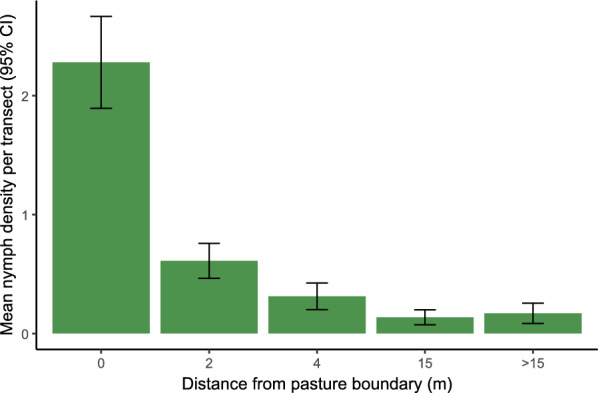
Mean nymph density (nymphs per transect, averaged across all farms) by distance from pasture boundary. Error bars represent 95% confidence intervals

A total of 1701 questing ticks were collected over the study period. Overall, ticks were found on 483 transects (20.3%). Nymphs were the most common life stage, comprising 91.3% of the total ticks collected (*n* = 1553), present in 461 (19.4%) transects, followed by adult males (*n* = 91, 5.3%), present in 66 (2.8%) transects and adult females (*n* = 57, 5.4%), present in 47 (2.0%) transects. The maximum number of nymphs recorded in a single transect was 36. Across all transects in the study, the mean nymph density was 0.65 nymphs per transect (SD 2.19). At the pasture boundary (0 m distance from pasture boundary, *n* = 432 transects across all farms), the overall mean nymph density was 2.28 nymphs per transect (SD 4.1, max = 36), with mean densities declining at increasing distances into the pasture (Fig. 3). In total, ticks were collected from 64 of the 72 sampled pastures (88.9%), with 75% of all ticks collected from 12 (32.4%) pastures. The maximum number of ticks collected in a single pasture was 104. Larvae were occasionally observed and recorded as present or absent but occurred at very low frequency and were therefore not included in quantitative analyses.

### Environmental variables

Grass was the dominant vegetation type recorded in 92% of transects across all farms, with herbaceous/wildflowers (4.1%), bare ground (1.7%), bramble/fern (1.5%), and rushes (0.7%) less frequently observed. Hedgerows were recorded as a boundary feature at 81% of 0 m transects, visible fences at 61.9%, and ditches at 5.3%. More than one of these features was present at 48.1% of 0 m transects (*n* = 208). The dominant habitat within 10 m on the opposite side of the boundary at 0 m transects was pasture (47.5%), followed by trees (26.3%), track/road (18.3%), scrubland (4.2%), and water (3.7%).

### Environmental factors influencing tick presence at transect level

The presence of questing ticks at the transect level was significantly associated with distance from the pasture boundary, vegetation height, and the proportion of woodland within a 50 m pasture buffer (Table 2). The probability of detecting ticks decreased with increasing distance from the boundary (−0.14 ± 0.01, *P* < 0.001), increased with vegetation height (0.06 ± 0.01, *P* < 0.001), and increased with proportion of woodland within a 50 m buffer around the entire pasture (0.05 ± 0.01, *P* < 0.001); values are model estimates ± SE. Vegetation density, dominant vegetation type, number of cow dung piles, and saturation deficit did not have significant impact on presence of questing ticks at transect level (i.e., were dropped during the model selection process, without an increase in AIC of ≥ 2; see Additional file 1). Random effects indicated additional variation at the farm (SD 0.17) and pasture (SD 1.20) levels. The final GLMM showed good model performance, having a conditional AUC of 0.900 (95% CI 0.886–0.915) and a marginal AUC of 0.833 (95% CI 0.814–0.852), with a maximum TSS of 0.66.

**Table 2:** Final binomial GLMM results for the presence of questing ticks (nymphs and adults) across all transects

Fixed effects	Estimate ± SE	*z*-Value	*P*-value	Delta AIC
Intercept	−2.84 ± 0.29	−9.87	< 0.001	–
Vegetation height	0.06 ± 0.01	9.60	< 0.001	34.4
Distance from pasture boundary	−0.14 ± 0.01	−13.68	< 0.001	241.4
Woodland within 50 m pasture buffer	0.05 ± 0.01	5.34	< 0.001	25.2
Random effects	Variance ± SD			
Farm	0.03 ± 0.17	–	–	–
Pasture	1.44 ± 1.20	–	–	–

Fixed-effect estimates (logit scale) with standard error (SE), *z*, *P*, and delta AIC from the final model; random-effect variances and standard deviation (SD) are reported for farm and pasture. Predictors were retained on the basis of minimization of AIC; *P*-values are reported for inference only

### Environmental factors influencing nymph density at pasture boundaries

Nymph density in transects located 0 m from pasture boundaries was positively associated with the proportion of woodland within a 50 m boundary buffer (3.87 ± 0.79, *P* < 0.001) and to a lesser extent with boundaries adjacent to water (1.21 ± 0.38, *P* = 0.001), although water was present in only seven pastures sampled, which may limit the strength of this association (Table 3). Boundaries adjacent to track/road showed a marginally negative impact on nymph density (–0.56 ± 0.31, *P* = 0.07), while scrubland and tree-adjacent habitats were not significantly different in impact from pasture. Vegetation height, vegetation density, presence of a hedgerow, and dung piles were not significant predictors of nymph density. Random effects indicated additional unmeasured factors affecting nymph density at the farm (SD 0.53), pasture (SD 0.70), and boundary (SD 0.79) levels. The final GLMM explained 27.2% of the variance in nymph density through fixed effects alone (marginal *R*^2^), and 78.4% when random effects were included (conditional *R*^2^; lognormal method). Prediction error was lower for the full model (conditional RMSE = 2.12) compared with fixed effects alone (marginal RMSE = 3.80). Interaction terms between the proportion of woodland within a 50 m boundary buffer and adjacent habitat type were also evaluated to test whether the effect of nearby woodland varied across immediate habitat contexts. Inclusion of these interactions did not substantially improve model fit (ΔAIC < 2), and increased marginal *R*^2^ only slightly (from 0.26 to 0.30), while conditional *R*^2^ remained almost unchanged (0.74–0.80). These terms were therefore not retained in the final model (Additional file 1).

**Table 3:** Final negative binomial GLMM results for nymph density at pasture boundaries (0 m transects)

Final GLMM (fixed effects)	Estimate ± SE	*z*-Value	*P*-value	Delta AIC
Intercept	−0.76 ± 0.32	−2.38	0.02	–
Vegetation height	−0.01 ± 0.01	−1.61	0.11	–
Vegetation density	0.01 ± 0.01	1.60	0.11	–
Hedgerow	0.30 ± 0.20	1.49	0.14	–
Woodland within 50 m boundary buffer	3.87 ± 0.79	4.92	< 0.001	26.2
Cattle dung piles	0.12 ± 0.08	1.47	0.14	–
Adjacent habitat: scrubland	0.16 ± 0.47	0.33	0.74	–
Adjacent habitat: track/road	−0.56 ± 0.31	−1.81	0.07	–
Adjacent habitat: water	1.21 ± 0.38	3.12	0.001	7.8
Adjacent habitat: trees	0.19 ± 0.23	0.80	0.42	–
Random effects	Variance ± SD			
Farm	0.28 ± 0.53	–	–	–
Pasture	0.48 ± 0.70	–	–	–
Boundary	0.62 ± 0.79	–	–	–

Fixed-effect estimates (log scale) with SE, *z*, *P*, and delta AIC from the final GLMM are shown with random-effect variances for farm, pasture, and boundary. Predictors were retained on the basis of minimization of AIC; *P*-values are reported for inference only. Reference level for adjacent habitat = pasture

### Cattle pathogen prevalence and associations with tick abundance

Broad scale correlations at the farm level between herd-level (adult cows) *B. divergens* and *A. phagocytophilum* prevalence and the farm-level mean tick abundance at 0 m transects or woodland cover within 50 m buffers were weak and not statistically significant. Spearman rank correlation coefficients (*ρ*) ranged from −0.38 to 0.47. See Additional file 2.

## Discussion

This is the first study to examine the landscape factors driving the abundance of the key tick vector, *I. ricinus*, within cattle grazing systems in the UK. By distinguishing the relative contributions of pasture-level vegetation and microclimate from those of ecotonal and broader habitat composition, it establishes a foundation for understanding how environmental structure influences tick hazard in livestock systems in regions where tick-borne disease is already established. By identifying the habitats and landscape features that sustain high tick densities, the findings provide a critical first step in anticipating how tick-borne disease risks may shift under environmental and climate policies across the UK and European Union that promote woodland expansion, habitat restoration, and landscape connectivity. As farms were selected to represent endemic systems, the results are intended to explain spatial variation in tick hazard within affected grazing landscapes rather than processes governing initial tick establishment or spread into previously unaffected farms.

Tick sampling was conducted during a single period in late spring–early summer, coinciding with peak nymphal activity in the UK, and therefore represents a cross-sectional snapshot of tick abundance at that time. While absolute tick densities are expected to vary across seasons and years, the use of a standardized sampling protocol applied consistently across all farms during the same sampling period supports robust comparison of relative tick hazard among pastures and boundary types within the study region. Tick distribution was highly heterogeneous, with strong spatial aggregation both between and within pastures. Densities were concentrated at pasture margins and declined sharply into open pastures, consistent with patterns reported across European agricultural landscapes [15, 28–30]. The limited influence of several pasture- and transect-level vegetation and environmental variables suggests that fine-scale conditions within pastures play a relatively minor role in structuring tick abundance compared with edge position itself. This sharp edge-to-field gradient is compatible with either improved survival/questing in ecotonal margins and/or short range spillover from adjacent woodland habitat. Understanding mechanisms would require further studies, and measuring host, habitat, and microclimate across a gradient from the pasture edge into the adjacent woodland habitat. The proportion of woodland cover within 50 m buffers around pasture boundaries and entire pastures were the dominant predictors of tick abundance, whereas hedgerow boundaries and immediate adjacency to tree habitats within 10 m of pasture boundaries showed no association. This contrasts with studies in UK arable systems and French cattle pastures where hedgerows and tree cover along pasture perimeters have been associated with increased tick presence or abundance [15, 28, 30].

Hedgerows bordered most pasture edges in our study system (81%), reducing statistical power to detect independent effects. In addition, the hedgerows were recorded only as a binary variable, which did not capture structural variation such as width, density, and composition, known to influence tick populations [28, 30]. In hedgerow-dominated dairy landscapes, such binary measures may therefore provide poor indicators of vegetated microsites used by tick hosts. Although more detailed hedgerow metrics derived from remote sensing have been developed for use at national scales, comparison with field observations indicated that these did not reliably represent hedgerow characteristics at the scale of individual pasture boundaries in this study area.

The absence of an effect of adjacency to tree habitats within 10 m of pasture boundaries suggests that processes influencing *I. ricinus* distribution in grazing pastures operated across broader spatial scales than local boundary features. Woodland extent in 50 m buffers captures local landscape composition and the availability of suitable woodland habitat that are known to influence tick populations [13, 62]. Modeling studies suggest woodlands are a source of ticks for pastures, with migration across ecotones required to sustain the presence of ticks in pastures [63]. While evidence for deer transporting ticks into pastures is mixed [64], regular reported deer sightings in and around cattle pastures in our study are consistent with this potential mechanism. Small mammals also contribute to tick abundance at the woodland–ecotone–pasture interface, although their dispersal role is complex and species-specific [17].

While hedgerows can contribute to habitat connectivity and facilitate host movement between woodland and pastures, they were not detected as important habitats for sustaining tick populations along pasture boundaries in our study landscape. This has implications for how agri-environment schemes that promote hedgerow creation or maintenance are interpreted, as hedgerow presence alone may not capture the structural or contextual features most relevant to tick hazard. It is therefore important to study hedgerow effects on tick abundance and hazard in other farmland contexts, sampling across different boundary types and integrating more detailed metrics of hedgerow structure.

At the broader landscape scale, woodland cover within 50 m buffers was used as a pragmatic proxy for the local availability of woodland habitat adjacent to pasture edges, rather than a detailed measure of woodland configuration or quality. We did not quantify woodland patch size, age, understory structure, or woodland type at the scale of individual pasture boundaries, and these attributes may further influence host use and the microclimatic conditions that support *I. ricinus*. In addition, tick densities within adjacent woodland were not measured and may mediate the extent to which woodland acts as a source of ticks for nearby pasture margins. Future work integrating woodland structural metrics, host activity data, and paired sampling within woodland and pasture-edge habitats would help resolve how woodland composition and configuration translate into tick hazard in grazing systems.

By contrast, adjacency to water predicted higher nymph densities in boundary transects. To our knowledge, this is the first study to examine the influence of local water bodies on tick abundance in UK grazing pastures. A study in a European landscape reported *I. ricinus* abundance to be high along rivers in wet, humid canopies supporting dense undergrowth with high relative humidity [62]. Riparian zones support a high proportion of wildlife [65], increasing opportunities for host–vector contact and tick dispersion into pasture edges.

Our results indicate that cattle exposure to ticks is likely to be concentrated at pasture edges, particularly those bordering woodland and water bodies. Restricting cattle access to these areas could theoretically reduce exposure to ticks, but these areas also provide shade, shelter, and valuable grazing, creating welfare and economic tradeoffs [66]. Furthermore, complete avoidance of tick exposure is neither feasible nor advised in pasture-based systems within endemic regions. Low-level exposure to infected ticks is thought to support protective immunity to *B. divergens*, and controlled exposure remains a recommended strategy for herd management in endemic areas [24, 67]. However, key uncertainties remain regarding the spatial distribution of infected ticks within pastures (e.g., whether hazard mirrors patterns in tick abundance), the development and persistence of protective immunity, and how controlled exposure could be implemented at farm level.

Tick pathogen data were not available in this study, however, published studies report *B. divergens* prevalence in questing ticks to be low across UK and European landscapes (generally 0–4%) [68–71], while *A. phagocytophilum* prevalence is higher and more variable, ranging from 0% to approximately 20% [68, 72, 73]. Consequently, while questing nymph density is a useful proxy for cattle exposure, due to the diverse hosts and pathways involved in transmission of *B. divergens* and *A. phagocytophilum* [74], infection risk could remain low or inconsistent even in habitats with high nymph densities, limiting the predictive value for assessing farm-level risk. No significant associations were detected between farm-level tick abundance or woodland cover and pathogen prevalence in cattle. These correlations are interpreted cautiously, as inference is complicated by aggregation across multiple levels, including individual animal PCR results summarized as herd-level prevalence, tick counts averaged to mean abundance per farm, and landscape variables averaged across pastures. The absence of strong associations likely reflects a combination of limited statistical power and the lag between tick exposure and detectable infection in cattle, which may depend on exposure accumulated across multiple grazing seasons, pathogen prevalence within tick populations, host immunity, and farm management practices such as acaricide use and grazing patterns that were not captured in this study.

Our findings suggest potential unintended consequences of agri-environment policies that promote woodland expansion and connectivity in farmed landscapes. In our study, greater woodland extent within grazing systems was associated with higher tick abundance at pasture edges, which may increase tick exposure at woodland–pasture interfaces in landscapes where ticks are already present. Similar links between tick abundance or tick-borne pathogen ecology and landscape context in grazed systems have been reported elsewhere in Europe, including associations with woodland/edge habitats and environmental determinants of cattle infection [47, 75, 76]. However, tick presence and abundance represent one component of exposure hazard and should not be equated directly with disease risk, which additionally depends on pathogen prevalence, host exposure and immunity, and temporal dynamics that were not the primary focus of this study. Multiyear studies integrating tick and cattle sampling, pathogen screening, host activity, and detailed farm management data are required to determine whether landscape changes driven by agri-environment schemes will contribute to measurable increases in tick-borne disease risk, and to inform evidence-based recommendations for disease control at farm level. In the interim, strengthened farm surveillance of tick-borne diseases will be essential to guide management and policy decisions [77].

Finally, the implications of this study extend beyond livestock health. High nymph densities in ecotonal habitats within grazing pastures also pose a risk to humans, given that *I. ricinus* is the primary UK vector of *Borrelia burgdorferi* sensu lato, the causative pathogen of Lyme disease, and tick-borne encephalitis virus, both detected in southern England [78, 79]. Farmers and recreational users of farmland may therefore be exposed to infected nymphs at pasture margins, reinforcing the continued importance of awareness campaigns promoting personal protective measures.

## Conclusions

This study provides the first systematic analysis of the influence of local landscape features on tick hazard in UK dairy systems. Tick densities were highly aggregated across pastures and in ecotonal habitats. Woodland cover was the dominant predictor of tick abundance, and adjacency to water bodies also contributed to elevated nymph densities at pasture edges. Hedgerows were not significant predictors of nymph density in our study farms. In a policy context, the findings highlight a critical tension. Woodland creation and connectivity are central to EU and UK environmental targets, delivering clear biodiversity and climate benefits, yet woodland established adjacent to grazed pastures could inadvertently increase livestock exposure to ticks. Translating ecological risk factors into policy recommendations and farm-level disease control interventions is challenging. Further research should prioritize longitudinal, multiseason studies integrating tick abundance, pathogen prevalence, and livestock exposure to improve understanding of how landscape change may influence disease risk over time. In parallel, cross-sector engagement spanning agriculture, land management, environmental policy, and public health will be essential to co-develop evidence-based recommendations that reconcile biodiversity and climate objectives with the protection of livestock and public health.

## Supplementary Information

Additional file 1.

## Data Availability

The data supporting the conclusions of this article are included within the article and its additional files.
