# Supplementary material for: Environmental drivers of tick density in UK dairy farms: implications for livestock health and agri-environment policy
Source: Parasit Vectors. 2026 Mar 31;19:205. doi: 10.1186/s13071-026-07345-w (PMC13159359; doi:10.1186/s13071-026-07345-w)
Supplement: Supplementary file 1 — Additional file 1. [file 13071_2026_7345_MOESM1_ESM.docx]

**Supplementary methods: Tick species identification and molecular confirmation**

To further assess if other Ixodes species were present among the sampled nymphs, a probability analysis was carried out to calculate a sample size for molecular screening using 16S PCR and sequencing N=log(1-P)/log (1-r) [1] . To determine the sample size required to ensure a high level of accuracy, a high probability of detection (P) of 0.99 and a conservative rate of identifying another species (r) of 0.11 was set. This provided an estimate of a sample size of at least 40 required to detect a tick species other than I*. ricinus*. Based on this calculation, a random subset of field collected nymphs from across all farms (n = 144) were screened by PCR, with all valid results (n = 123) confirming *I. ricinus*.

**Additional file 1: Model selection results**

**Model selection**

Backward stepwise selection was performed with drop1(..., test = "Chisq"). “AIC_after” is the AIC of the updated model; “ΔAIC” is the change from the previous step. *LRT* and *p* are the likelihood-ratio test statistic and p-value comparing the reduced model to the previous model. The final model is the last step, where removing any remaining term increased AIC.

**Table S1** Backward stepwise model selection for tick presence across all transects (binomial GLMM).

| **Step** | **Model** | **Removed term** | **AIC_after** | **ΔAIC** | **LRT** | **p_value** |
| --- | --- | --- | --- | --- | --- | --- |
| 0 | model_1 (full) | (none) | 1667.624 |  |  |  |
| 1 | model_1a | dom.veg | 1663.443 | -4.181 | 3.82 | 0.43 |
| 2 | model_1b | SD | 1661.766 | -1.677 | 0.32 | 0.57 |
| 3 | model_1c | veg_density | 1661.206 | -0.560 | 1.44 | 0.23 |
| 4 | model_1d (final) | cow_piles | 1660.800 | -0.406 | 1.59 | 0.21 |

**Table S2** Variance Inflation Factors (VIF) for fixed effects included in the GLMM assessing tick presence/absence. VIF values < 3 were considered indicative of acceptable collinearity.

| **Predictor** | **VIF** |
| --- | --- |
| Vegetation height | 2.14 |
| Vegetation density | 1.9 |
| Distance from pasture boundary | 1.05 |
| Wood 50m pasture buffer | 1.02 |
| Dominant vegetation type | 1.31 |
| Saturation deficit | 1 |
| Number of cow dung piles | 1.01 |

**Table S3** Backward stepwise model selection for nymph density at pasture boundaries (0 m) (negative binomial GLMM).

| **Step** | **Model** | **Removed term** | **AIC_after** | **ΔAIC** | **LRT** | **p_value** |
| --- | --- | --- | --- | --- | --- | --- |
| 0 | neg_bin_model1 (full) | (none) | 1436.23 |  |  |  |
| 1 | model_1a | dom.veg | 1431.004 | -5.226 | 2.77 | 0.60 |
| 2 | model_1b | SD | 1429.479 | -1.525 | 0.48 | 0.49 |
| 3 | model_1c | Visible.fence | 1428.624 | -0.855 | 1.15 | 0.28 |
| 4 | model_1d (final) | Ditch | 1428.515 | -0.109 | 1.89 | 0.17 |

**Table S4** Variance Inflation Factors (VIF) for fixed effects included in the GLMM assessing nymph density. VIF values < 3 were considered indicative of acceptable collinearity.

| **Predictor** | **VIF** |
| --- | --- |
| Vegetation height | 1.81 |
| Vegetation density | 1.55 |
| Ditch | 1.06 |
| Visible fence | 1.14 |
| Hedgerow | 1.12 |
| Dominant vegetation type | 1.58 |
| Wood 50 m boundary buffer | 1.49 |
| Saturation defecit | 1.03 |
| Number of cow dung piles | 1.05 |
| Adjacent habitat type | 1.77 |

**Additional file 2: Cattle pathogen prevalence and correlation analysis**

**Table S5** Cattle pathogen prevalence with 95% confidence intervals (CI) and mean tick abundance with standard deviation (SD) (calculated as the average number of ticks per transect at 0 m from pasture boundaries within sampled grazing pastures on each farm).

| **Farm** | **Mean tick abundance (SD)** | ***A. phagocytophilum*** | ***B. divergens*** |
| --- | --- | --- | --- |
|  |  | **% prevalence**  **(95% CI)** | **% prevalence**  **(95% CI)** |
| 1 | 2.17 (4.96) | 24 (11.5-43.4) | 4 (0.7-19.5) |
| 2 | 3.83 (4.09) | 10 (4.9-19.2) | 0 (0.0-5.2) |
| 3 | 2.56 (5.52) | 4 (1.1-13.5) | 2 (0.4-10.5) |
| 4 | 3.14 (4.67) | 8.3 (3.9-17.0) | 0 (0.0-5.1) |
| 5 | 1.61 (1.90) | 7.9 (3.7-16.2) | 0 (0.0-4.8) |
| 6 | 5.00 (4.55) | 15.8 (9.3-25.6) | 0 (0.0-4.8) |
| 7 | 2.56 (4.43) | 4.7 (1.6-12.9) | 0 (0.0-5.7) |
| 8 | 1.11 (2.43) | 0 (0.0-6.0) | 0 (0.0-6.0) |
| 9 | 0.81 (1.17) | 0 (0.0-6.0) | 0 (0.0-6.0) |
| 10 | 1.56 (2.82) | 38.7 (27.6-51.2) | 0 (0.0-5.8) |
| 11 | 1.58 (2.96) | 8.5 (3.7-18.4) | 0 (0.0-6.1) |
| 12 | 4.11 (7.21) | 7.1 (3.1-15.7) | 0 (0.0-5.2) |

**Table S6** Spearman rank correlation coefficients (ρ) describing farm-level associations between cattle pathogen prevalence in adult cattle, mean questing tick abundance at pasture boundaries (0 m transects), and woodland cover within 50 m buffers.

| Variable 1 | Variable 2 | Spearman’s ρ | p-value |
| --- | --- | --- | --- |
| AP prevalence | BD prevalence | 0.108 | 0.739 |
| AP prevalence | Mean tick abundance (0 m) | 0.295 | 0.352 |
| AP prevalence | Wood 50m pasture buffer | 0.081 | 0.803 |
| AP prevalence | Wood 50 m boundary buffer | 0.018 | 0.957 |
| BD prevalence | Mean tick abundance (0 m) | 0.024 | 0.94 |
| BD prevalence | Wood 50m pasture buffer | -0.344 | 0.274 |
| BD prevalence | Wood 50 m boundary buffer | -0.375 | 0.23 |
| Mean tick abundance (0 m) | Wood 50m pasture buffer | 0.466 | 0.127 |
| Mean tick abundance (0 m) | Wood 50m pasture buffer | 0.069 | 0.832 |
| Wood 50m pasture buffer | Wood 50 m boundary buffer | 0.814 | 0.00127 |

References

1. Gu, W. and R.J. Novak, *Detection probability of arbovirus infection in mosquito populations.* The American journal of tropical medicine and hygiene, 2004. **71**(5): p. 636-638.
